# Neoadjuvant Therapy for Extrahepatic Biliary Tract Cancer: A Propensity Score-Matched Survival Analysis

**DOI:** 10.3390/jcm12072654

**Published:** 2023-04-02

**Authors:** Junya Toyoda, Kota Sahara, Tomoaki Takahashi, Kentaro Miyake, Yasuhiro Yabushita, Yu Sawada, Yuki Homma, Ryusei Matsuyama, Itaru Endo, Timothy M. Pawlik

**Affiliations:** 1 Department of Gastroenterological Surgery, Yokohama City University School of Medicine, Yokohama 236-0004, Japan; 2 Department of Surgery, Division of Surgical Oncology, The Ohio State University Wexner Medical Center and James Comprehensive Cancer Center, Columbus, OH 43210, USA

**Keywords:** neoadjuvant therapy, biliary tract cancer, gallbladder cancer, extrahepatic cholangiocarcinoma

## Abstract

**Background:** Although surgery is the mainstay of curative-intent treatment for extrahepatic biliary tract cancer (EBTC), recurrence following surgery can be high and prognosis poor. The impact of neoadjuvant therapy (NAT) relative to upfront surgery (US) among patients with EBTC remains unclear. **Methods:** The Surveillance, Epidemiology, and End Results (SEER) databases was utilized to identify patients who underwent surgery from 2006 to 2017 for EBTC, including gallbladder cancer (GBC) and extrahepatic cholangiocarcinoma (ECC). Trends in NAT utilization were investigated, and the impact of NAT on prognosis was compared with US using a propensity score-matched (PSM) analysis. **Results:** Among 6582 EBTC patients (GBC, *n* = 4467, ECC, *n* = 2215), 1.6% received NAT; the utilization of NAT for EBTC increased over time (P_trend_ = 0.03). Among patients with lymph node metastasis, the lymph node ratio was lower among patients with NAT (0.18 vs. 0.40, *p* < 0.01). After PSM, there was no difference in overall survival (OS) and cancer-specific survival (CSS) among patients treated with NAT versus US (5-year OS: 24.0% vs. 24.6%, *p* = 0.14, 5-year CSS: 38.0% vs. 36.1%, *p* = 0.21). A subgroup analysis revealed that NAT was associated with improved OS and CSS among patients with stages III–IVA of the disease (OS: HR 0.65, 95%CI 0.46–0.92, *p* = 0.02, CSS: HR 0.62, 95%CI 0.41–0.92, *p* = 0.01). **Conclusions:** While NAT did not provide an overall benefit to patients undergoing surgery for EBTC, individuals with advanced-stage disease had improved OS and CSS with NAT. An individualized approach to NAT use among patients with EBTC may provide a survival benefit.

## 1. Introduction

Biliary tract cancer (BTC) is a rare, heterogeneous malignancy arising from the epithelial cells of the bile ducts and gallbladder [1,2]. Specifically, BTC refers to a spectrum of invasive adenocarcinomas, including gallbladder cancer (GBC) and cholangiocarcinoma (cancers arising in the intrahepatic, perihilar, or distal biliary tree) [3]. The incidence of GBC is approximately 2.0 per 100,000, with gallstones and chronic infection of the gallbladder being the most important risk factors for the development of GBC [4]. The incidence of extrahepatic cholangiocarcinoma (ECC), including hilar cholangiocarcinoma (HCC) and distal cholangiocarcinoma (DCC), is 0.5 to 1.1 per 100,000, with cirrhosis, hepatitis B and C infections, and primary sclerosing cholangitis being known risk factors [4]. Due to the dismal prognosis of GBC and ECC, standard treatment options are limited and surgery is the mainstay of curative-intent treatment [3]. In the clinical setting, most patients are not candidates for surgery at the time of diagnosis, and recurrence after resection is common [5]. Patients with advanced GBC and ECC who undergo resections also have a poor 5-year prognosis ranging from 10–25% and 2–30%, respectively [6,7].

In terms of perioperative treatment of BTC, adjuvant therapy has been recommended especially for patients at high risk of recurrence (i.e., lymph node metastasis) based on results of the BILCAP trial [5,7,8]. In contrast, the role of neoadjuvant therapy (NAT) for EBTC has not been defined. The rationale for NAT includes the eradication of micro-metastases, reduction in primary tumor size, optimization of patient selection for surgery, and ultimately improved survival [2]. In turn, NAT has become commonly used to treat many types of gastrointestinal malignancies, such as esophageal, gastric, pancreatic, and rectal cancers [9,10,11,12,13,14]. The objective of the present study was to characterize the impact of NAT on the prognosis of patients with EBTC. In particular, we sought to assess trends in NAT utilization, as well as the impact of NAT on postoperative survival outcomes among patients with GBC and ECC using a national population-based database.

## 2. Methods

### 2.1. Dataset and Study Population

The Surveillance, Epidemiology, and End Results (SEER) database is a large public cancer database available in the U.S. GBC and ECC patients who underwent surgery from 2010 to 2017 were identified in the dataset of “SEER Research Plus Data 17 Registries, No 2021 Sub (2000–2019)”. The SEER program collects and publishes data on cancer incidence and survival population-based cancer registries in 22 U.S. geographic areas (available at https://seer.cancer.gov/registries/list.html. Accessed on 1 February 2023). Patient data were extracted based on the International Classification of Disease for Oncology 3rd edition (ICD-O-3) site codes related to primary sites (C23.9, C24.0) [15,16]. Demographic and clinical data included age at the time of diagnosis, sex, race, year of diagnosis, tumor characteristics (i.e., primary tumor site, AJCC 7th classification, histological grade), as well as the use of neoadjuvant treatment. In addition, survival analyses were performed based on cause-specific death classifications. The code of “Dead” (attributable to this cancer dx) was used to identify deaths due to GBC and ECC, whereas “other codes for death” (dead of other cause and N/A not first tumor) defined mortality from other causes. Patients with distant metastases, individuals without data on the AJCC 7th classification, perioperative chemotherapy and radiotherapy, as well as patients who did not have postoperative follow-up within 3 months of surgery were excluded (Supplementary Figure S1). NAT included preoperative chemotherapy, regardless of radiation use.

### 2.2. Statistical Analysis

The study cohort was categorized into NAT and upfront surgery (US) groups. Continuous variables were compared with the Mann–Whitney U or Kruskal–Wallis tests, as appropriate. Categorical variables were compared with the χ^2^ or Fisher’s exact tests, as appropriate. The Cochran–Armitage tendency test was used to perform a trend analysis to assess the changes in the proportion of patients undergoing NAT over time [17]. Statistical significance was assessed at α = 0.05. To balance the potential confounding variables among patients who did versus did not receive NAT, a 1:4 (NAT: US) nearest neighbor propensity score-matching (PSM) test was performed [18]. The cohorts were matched based on multiple variables: age, gender, race, year of diagnosis, cancer site, AJCC 7th T and N stages, histologic tumor grade, and receipt of adjuvant therapy. Subsequently, overall survival (OS) and cancer specific survival (CSS) were calculated and displayed using Kaplan–Meier curves and evaluated using the log-rank test. Cox regression analysis was used for overall and subgroup analyses to examine whether NAT prolonged OS and CSS. All statistical analyses were performed using JMP statistical package version 15 (SAS Institute Inc., Cary, NC, USA) and R version 4.2.0 (Vienna, Austria).

## 3. Results

### 3.1. Patients, Tumor, and Treatment Characteristics

**Figure 1:**
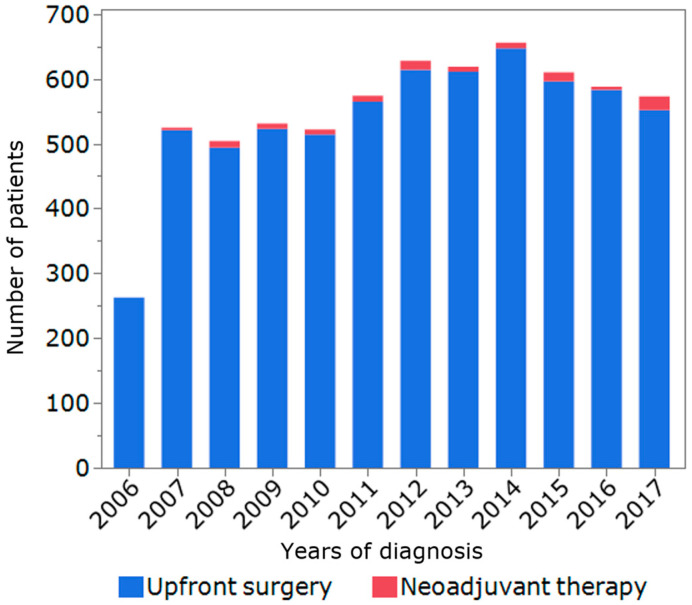
Trends in the number of patients with extrahepatic biliary tract cancer in the SEER registry.

A total of 6582 patients met inclusion criteria and were included in the final analytic cohort (GBC, *n* = 4467, 67.9%, ECC, *n* = 2215, *n* = 32.1%). Among 108 patients who received NAT (1.6%), the majority received neoadjuvant chemotherapy only (*n* = 80, 74.1%), while 28 patients (25.9%) received neoadjuvant chemoradiation therapy. The proportion of patients with NAT gradually increased over time (2006–2009: *n* = 22, 1.2%; 2010–2013: *n* = 39, 1.7%; 2014–2017: *n* = 47, 2.1%, P_trend_ = 0.03) (Figure 1). Compared with patients who underwent US, individuals who received NAT were younger (65 years old, IQR 57–71 vs. 70 years old, IQR 61–78, *p* < 0.01), male (*n* = 59, 54.6% vs. *n* = 2694, 41.6%, *p* < 0.01), more likely to have ECC (*n* = 70, 64.8% vs. *n* = 2045, 31.6%, *p* < 0.01), T3/T4 disease (*n* = 69, 63.9% vs. *n* = 2452, 37.9%, *p* < 0.01), lymph node metastasis (*n* = 55, 50.9% vs. *n* = 2039, 31.5%, *p* < 0.01), and stages III/IV (*n* = 62, 57.4% vs. *n* = 2653, 40.3%, *p* < 0.01). In terms of lymphadenectomy, the number of lymph nodes examined was higher among the patients treated with NAT (7, IQR 3–15 vs. 4, IQR 1–11, *p* < 0.01). Among the subset of individuals who had lymph node metastasis, the lymph node ratio (LNR) was lower in the NAT group (0.18, IQR 0.10–0.50 vs. 0.40, IQR 0.17–1.00, *p* < 0.01). Of note, there was no difference in the use of adjuvant therapy among patients who received NAT versus individuals who underwent US (*n* = 46, 42.6%, vs. *n* = 2794, 43.2%, *p* = 0.91) (Table 1).

### 3.2. Impact of NAT on Overall Survival before and after PSM

**Figure 2:**
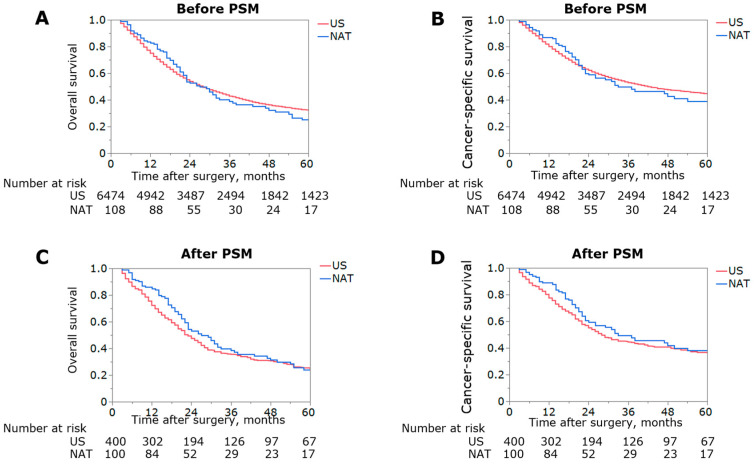
Kaplan–Meier curves depicting overall (**A**,**C**) and cancer-specific survival (**B**,**D**) among patients with extrahepatic biliary tract cancer before and after propensity score matching, respectively.

With a median follow-up of 26 months (IQR 12–54), median and 5-year OS for the entire cohort were 28 months (IQR: 13–90) and 32.0%, respectively. When stratified by receipt of NAT, patients treated with NAT had similar OS and CSS versus patients who underwent US (median OS: 27 vs. 28 months, 5-year OS: 25.0% vs. 32.0%, *p* = 0.67, median CSS: 33 vs. 42 months, 5-year CSS: 38.9% vs. 44.6%, *p* = 0.62) (Figure 2A,B). On multivariate analysis, NAT was not associated with improved OS (HR 0.86, 95%CI 0.68–1.09, *p* = 0.22) or CSS (HR 0.84, 95%CI 0.64–1.10, *p* = 0.21) (Supplementary Table S1).

To minimize any potential confounding, 1:4 PSM was utilized to create two matched cohorts of 100 and 400 patients. Following PSM, the patient cohorts were similar with regard to demographics and tumor characteristics (Supplementary Table S2). When stratified by receipt of NAT, 5-year OS and CSS remained similar among patients treated with NAT versus individuals who underwent US (median OS: 27 vs. 23 months, 5-year OS: 24.0% vs. 24.6%, *p* = 0.14, median CSS: 33 vs. 28 months, 5-year CSS: 38.0% vs. 36.1%, *p* = 0.21) (Figure 2C,D). For the multivariate analysis following PSM, while age <65, T1/2 and N0 were associated with improved OS (all *p* < 0.05), NAT remained not associated with better OS (HR 0.79, 95%CI 0.61–1.03, *p* = 0.08) or CSS (HR 0.78, 95%CI 0.58–1.07, *p* = 0.13) value (Table 2).

### 3.3. Subgroup Analysis of the Usefulness of NAT

**Figure 3:**
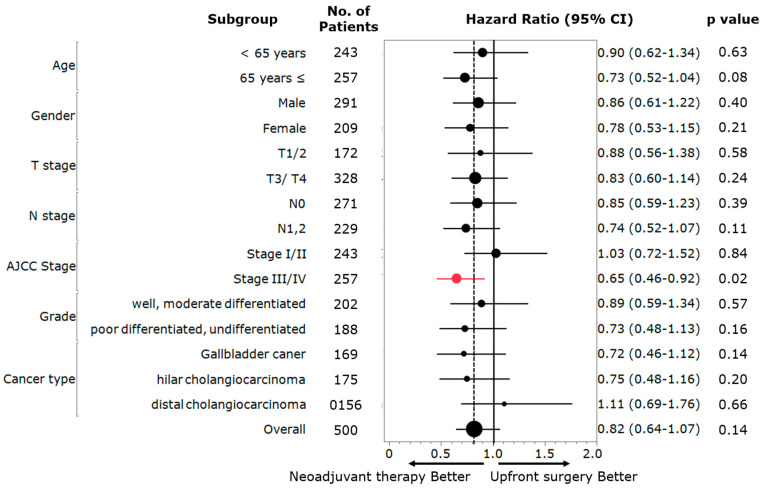
Subgroup analyses of overall survival in the PSM population. HR: hazard ratio; CI: confidence interval.

**Figure 4:**
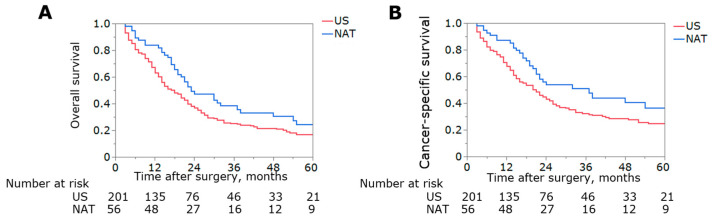
Kaplan–Meier curves depicting overall (**A**) and cancer-specific survival (**B**) stratified by use of NAT among patients with stages III/IVA of the disease.

To evaluate the association between NAT utilization and survival among different patient subgroups, Cox regression models using the PSM cohort were examined following stratification based on age, gender, T and N stages, AJCC 7th stage, tumor histological grade, and type of cancer. Notably, only patients with advanced-stage (i.e., stages III/IVA) disease derived a benefit from NAT (OS, HR 0.65, 95%CI 0.46–0.92, *p* = 0.02; CSS, HR 0.62, 95%CI 0.41–0.92, *p* = 0.01) (Figure 3, Supplementary Figure S2). Specifically, 5-year OS and CSS among patients at stages III/IVA of the disease who were treated with NAT had superior long-term outcomes compared with patients who underwent US (5-year OS: 24.4% vs. 16.8%, *p* = 0.01, 5-year CSS: 36.4% vs. 24.8%, *p* = 0.01) (Figure 4). The matched cohorts at stages III/IVA were comparable with regard to the demographics and tumor characteristics (Supplementary Table S3).

### 3.4. Sensitivity Analysis Excluding Patients with GBC

In light of the heterogeneity of tumor type across the entire cohort, sensitivity analyses were performed to examine the impact of NAT excluding patients with GBC (i.e., only individuals with hilar and distal cholangiocarcinomas). Among the 2215 patients with ECC, 70 (3.3%) received NAT. In a 1:4 PSM cohort comparison with similar demographics and tumor characteristics (Supplementary Table S4), 5-year OS and CSS were comparable among patients treated with NAT versus individuals who underwent US (median OS: 26 vs. 23 months, 5-year OS: 21.5% vs. 25.5%, *p* = 0.47, median CSS: 32 vs. 31 months, 5-year CSS: 34.9% vs. 37.4%, *p* = 0.64) (Supplementary Figure S3). In a subgroup analysis of patients with stages III/IVA of ECC, NAT improved long-term prognosis (OS: HR 0.53, 95%CI 0.30–0.92, *p* = 0.02; CSS: HR 0.55, 95%CI 0.29–1.03, *p* = 0.06).

## 4. Discussion

The natural history of BTC can be characterized as an aggressive disease course and numerous patients with BTC present with an advanced stage of the disease. Indeed, only 20% of BTC patients are eligible for surgery at the time of diagnosis, despite surgical resection being the only curative treatment option [5]. In fact, 5-year survival among patients with resected GBC and ECC ranges from 20–50%, even following an R0 resection [19,20]. Long-term prognosis is poor due to the high incidence of local and distant recurrences following resection [19,20]. In that context, adjuvant therapy for patients with BTC has been a topic of increased interest. BILCAP was a randomized phase III trial of adjuvant chemotherapy for patients with BTCs [8]. In this trial, 447 BTC patients were randomized to receive either adjuvant chemotherapy (i.e., capecitabine alone) or surgery alone. Of note, patients who received capecitabine had an improved OS (53 vs. 36 months) [8]. Based on these results, the American Society of Clinical Oncology (ASCO) Clinical Practice Guideline recommended that patients with resected BTCs should receive six months of postoperative adjuvant chemotherapy with capecitabine. Given the potential benefit of adjuvant therapy, the role of NAT among patients with BTCs has gained further interest; however, it remains ill-defined [21,22]. The present study is important because we specifically examined the impact of NAT on postoperative survival among patients with GBC and ECC using a large population database. NAT use among patients with EBCT was very low, although its utilization increased over the time periods examined. Following PSM, the use of NAT versus an US approach was not associated with an OS benefit (median OS: 27 vs. 23 months, respectively). However, in the subset of patients who presented with advanced EBCT disease, NAT was associated with both an OS and CSS benefit compared with US.

The proposed benefits of NAT are multifold. NAT may cytoreduce the tumor, thereby increasing the chance of an R0 resection. NAT may also allow for the earlier treatment of a micro-metastatic disease, as well as define the overall tumor biology. In turn, a NAT approach has become increasingly utilized in the treatment algorithm for many different types of cancers [21,23]. Over the last decade, a NAT approach has been widely embraced by clinicians treating patients with pancreatic adenocarcinomas [13]. Data from phase III trials have demonstrated better long-term outcomes among patients with pancreatic adenocarcinomas treated with NAT versus US [12,13]. In a one study, Utama et al. reported the benefit of NAT over US among patients with advanced intrahepatic cholangiocarcinomas (stages II and III) [18]. In contrast, our group previously noted no survival benefits of NAT among patients undergoing a resection of ampullary carcinomas versus US [24]. The role of NAT to treat patients with EBTCs has not been well-studied, with the results indicating the potential benefit of NAT being varied [20,25,26,27,28,29]. The reasons for these disparate findings are likely multifactorial and relate to the differences in the baseline characteristics of patients in the NAT versus US cohorts. In the present study, we utilized PSM to mitigate these baseline differences. Of note, while NAT was not associated with an OS or CSS benefit among patients in the entire cohort, NAT did provide a long-term prognostic benefit for patients with advanced-stage EBTC (Figure 4). Patkar et al. similarly reported that patients with advanced incidental GBC enjoyed a survival advantage with NAT (3-year OS, NAT: 59.9% vs. US: 32.3%) [30]. Therefore, the data suggest that a NAT approach should be considered for patients with EBTC who present with advanced disease.

The surgical management of EBTC involves both primary tumor resection and lymphadenectomy [31,32]. In fact, lymph node status has repeatedly been reported as one of the most powerful prognostic factors [33]. In particular, LNR, which is defined as the number of metastatic lymph nodes divided by the total number of lymph nodes examined, has been proposed as an accurate means to stratify patients with GBC and CCA relative to lymph node evaluations [34,35,36]. In the present study, LNR was able to stratify patients into three distinct prognostic groups relative to survival (median OS: LNR = 0 vs. 0 < LNR < 0.2 vs. 0.2 < = LNR, 54 vs. 24 vs. 19 months, *p* < 0.01). Interestingly, the median LNR was lower among patients treated with NAT compared with the US group. In turn, these data may suggest that NAT was able to downstage some patients who presented with lymph node metastasis. To this point, Hao et al. noted the downstaging of lymph node metastasis after neoadjuvant intraperitoneal and systemic chemotherapies for gastric carcinomas with peritoneal metastasis [37]. In a different study, other investigators reported a marked pathological response to lymph nodes following NAT that correlated with the pathological response of the primary tumor among patients with breast or colon cancers [38,39]. In addition, there may be a survival benefit for patients with lymph node downstaging, which may act as an additional prognostic marker in the comparative evaluation of differing systemic chemotherapy regimens in clinical trials [40].

The ABC-02 trial noted that doublet therapy with cisplatin plus gemcitabine (GC) was associated with an improved prognosis among patients with advanced BTC versus monotherapy with gemcitabine alone [20]. Since the reporting of this study, GC therapy has generally been accepted as the standard chemotherapy for patients with BTC [20]. Median progression-free survival with the use of GC was still only 8.0 months among patients with advanced BTC. In turn, triplet regimens have more recently been examined as potential systemic therapy, even in the preoperative treatment setting [41,42,43,44]. For example, KHBO-1401 MITSUBA was a randomized phase III study that demonstrated a higher response rate among patients treated with S-1 plus GC versus GC alone (OS: 41.5% vs. 15.0%, respectively) [44]. In a separate phase II trial, the efficacy of therapy with anti-PD-L1 and anti-CTLA4 combined with GC was examined [45]. Of note, median OS was 18–20 months among patients with unresectable or metastatic BTCs, which was considerably longer than the 8.3 months reported in the ABC-02 trial with GC alone. This regimen is under investigation at present in the neoadjuvant setting for resectable BTC (DEBATE trial) [45]. In addition, the GAIN trial is underway at present as a phase III clinical trial in Germany [4]. This study will examine whether induction GC therapy followed by a radical resection prolongs survival compared with radical surgery alone for cholangiocarcinoma or incidental GBCs.

The results of the present study should be interpreted in the context of several limitations. Due to its retrospective nature, the present study may be subject to selection bias. Specifically, the indications for neoadjuvant therapy are important; however, these indiciations could not be investigated in the present study due to the retrospective nature of the database. As in the GAIN trial, NAT may have been administered after cholecystectomy prior to the hepatectomy in the case of early GBC [4]. In contrast, patients with stages III/IVA BTCs likely received NAT for borderline resectable or unresectable conditions and underwent surgery after treatment with NAT. It is also possible that patients diagnosed with an unresectable disease at one facility may have undergone NAT and received treatment at another facility; whether subsequent resectablity was due to downsizing due to NAT versus simply a different opinion regarding operability by another surgeon could not be assessed. While minimizing the effect of the observed confounders, PSM does not adjust for any potential unmeasured confounders. In particular, the SEER database did not capture data on functional status (e.g., ECOG performance status, Karnofsky performance status, and activity level by Mets, etc.); in addition, information on chemotherapy regimens, response, and perioperative data (e.g., operative time, operative blood loss, complications after surgery, R0 resection rate, etc.) were not available for analysis. In addition, the SEER database collects data from population-based cancer registries covering approximately 47.9% of the U.S. As such, the results from the present study may not be necessarily generalizable to tertiary hepato-pancreato-biliary hospitals, as well as non-U.S. institutions. Given the heterogeneity of tumor behavior and treatment strategies, the effect of NAT should ideally be examined in separate cohorts of patients with GBCs or cholangiocarcinomas. However, due to the relatively rarity of each BTC subtype, most clinical trials on perioperative chemotherapy have included both GBCs and cholangiocarcinomas when examining BTCs [20,44,46]. Future studies should, however, strive to investigate whether NAT prolongs survival in patients with advanced-stage BTC separately among patients with GBC versus ECC.

In conclusion, the overall utilization of NAT for patients with EBTCs was very low, yet increased over the time periods examined. While NAT did not provide an overall benefit to all patients undergoing surgery for EBTC, individuals with advanced-stage disease had improved OS and CSS with NAT. An individualized approach to NAT use for EBTC may provide a survival benefit to certain subsets of patients. 

## Figures and Tables

**Table 1:** Patient characteristics by use of neoadjuvant therapy.

	All		Upfront Surgery		Neoadjuvant Therapy		
Variable	*n* = 6582	%, IQR	*n* = 6474	%, IQR	*n* = 108	%, IQR	*p* Value
Age, mean (IQR)	70	61–78	70	61–78	65	57–71	<0.01
Gender, *n* (%)							<0.01
Male	2753	41.8	2694	41.6	59	54.6	
Female	3829	58.2	3780	58.4	49	45.4	
Race, *n* (%)							0.06
White	5046	76.7	4954	76.5	92	85.2	
Black	638	9.7	633	9.8	5	4.6	
Others	882	13.4	872	13.5	10	9.3	
Unknown	16	0.2	15	0.2	1	0.9	
Type of cancer, *n* (%)							<0.01
Gallbladder cancer	4467	67.9	4429	68.4	38	35.2	
Hilar cholangiocarcinoma	1135	17.2	1095	16.9	40	37.0	
Distal cholangiocarcinoma	980	14.9	950	14.7	30	27.8	
Year of diagnosis, *n* (%)							0.18
2006–2009	1820	27.6	1798	27.8	22	20.4	
2010–2013	2342	35.6	2303	35.6	39	36.1	
2014–2017	2220	36.8	2373	36.6	47	43.5	
AJCC T stage, *n* (%)							<0.01
T1	1223	18.6	1209	18.7	14	13.0	
T2	2838	43.1	2813	43.4	25	23.1	
T3	2260	34.3	2205	34.1	55	50.9	
T4	261	4.0	247	3.8	14	13.0	
AJCC N stage, *n* (%)							<0.01
N0	4488	68.2	4435	68.5	53	19.1	
N1	2038	31.0	1986	30.7	52	48.1	
N2	56	0.8	53	0.8	3	2.8	
AJCC Stage, *n* (%)							<0.01
Stage I	1281	19.4	1265	19.5	16	14.8	
Stage II	2586	39.3	2556	39.5	30	27.8	
Stage III	2440	37.1	2391	36.9	49	45.4	
Stage IVA	275	4.2	262	4.1	13	12.0	
Tumor grade, *n* (%)							<0.01
Well	1028	15.6	1020	15.8	8	7.4	
Moderate	3010	45.7	2978	46.0	32	29.6	
Poor	2010	30.5	1977	30.5	33	30.6	
Undifferentiated	62	1.0	61	0.9	1	0.9	
Unknown	472	7.2	438	6.8	34	31.5	
Number of lymph nodes examined, median (IQR)	4	1–11	4	1–11	7	3–15	<0.01
Number of positive lymph nodes, N1 median (IQR)	1	1–3	1	1–3	1	1–2	0.15
Lymph node ratio, N1 median (IQR)	0.40	0.17–1.00	0.40	0.17–1.00	0.18	0.10–0.50	< 0.01
Neoadjuvant radiation, *n* (%)	28	0.4	-	-	28	25.9	
Adjuvant therapy, *n* (%)	2840	43.1	2794	43.2	46	42.6	0.91

IQR: interquartile range; AJCC: American Joint Committee on Cancer.

**Table 2:** Univariate and multivariate Cox regression analyses with OS and CSS among propensity score-matched patients with extrahepatic biliary tract cancer.

		Univariate Analysis			Multivariate Analysis		
Variables	Reference	HR	95% CI	*p*	HR	95% CI	*p*
**OS**							
Age ≥ 65	<65	1.31	1.07–1.60	<0.01	1.30	1.07–1.60	<0.01
Male	Female	0.95	0.77–1.28	0.64	1.00	0.81–1.23	0.99
White	Other	0.96	0.74–1.25	0.76	0.91	0.70–1.20	0.52
GBC	EHCC	1.13	0.92–1.40	0.24	1.18	0.94–1.47	0.14
Grade poor, undifferentiated	Well, moderate	1.25	1.00–1.57	0.04	1.15	0.92–1.45	0.21
T3/4	T1/2	1.84	1.47–2.28	<0.01	1.68	1.33–2.13	<0.01
N1/2	N0	1.54	1.26–1.88	<0.01	1.38	1.11–1.70	<0.01
Stages III/IVA	Stages I/II	1.52	1.24–1.86	<0.01	-	-	-
Neoadjuvant therapy	No	0.82	0.63–1.07	0.14	0.79	0.61–1.03	0.08
Adjuvant therapy	No	1.11	0.91–1.36	0.28	0.93	0.75–1.16	0.55
CSS							
Age ≥ 65	<65	1.06	0.83–1.34	0.63	1.05	0.83–1.33	0.66
Male	Female	0.86	0.91–1.46	0.22	0.92	0.72–1.18	0.54
White	Other	0.91	0.68–1.24	0.57	0.83	0.62–1.13	0.25
GBC	EHCC	1.23	0.96–1.57	0.10	1.29	1.00–1.67	0.04
Grade poor, undifferenced	Well, moderate	1.30	1.00–1.69	0.04	1.17	0.89–1.53	0.24
T3/4	T1/2	2.24	1.71–2.95	<0.01	2.05	1.53–2.74	<0.01
N1/2	N0	1.69	1.33–2.14	<0.01	1.45	1.13–1.85	<0.01
Stages III/IVA	Stages I/II	1.84	1.45–2.34	<0.01	-	-	-
Neoadjuvant therapy	No	0.82	0.61–1.12	0.22	0.78	0.58–1.07	0.13
Adjuvant therapy	No	1.21	0.95–1.53	0.11	0.97	0.76–1.25	0.85

OS: overall survival; CSS: cancer specific survival; GBC: gallbladder cancer; EHCC: extrahepatic cholangiocarcinoma.

## Data Availability

The data are publicly available via the Center for Medicare Services.

## Supplementary Materials

The following supporting information can be downloaded at: https://www.mdpi.com/article/10.3390/jcm12072654/s1. Figure S1: Flowchart for selection of patients with extrahepatic bile tract cancer included in the study cohort.; Figure S2: Subgroup analyses of cancer-specific survival in the PSM; Figure S3: Kaplan–Meier curves depicting overall (A) and cancer-specific survival (B) stratified by use of NAT among patients with extrahepatic cholangiocarcinomas. Table S1: Univariate and multivariate Cox regression analyses with OS and CSS among patients with extrahepatic biliary tract cancer before propensity score matching; Table S2: Patient characteristics after propensity score matching stratified by neoadjuvant therapy. Table S3: Patient characteristics after propensity score matching among patients with stages III/IVA of the disease; Table S4: ECC patient characteristics after propensity score matching stratified by neoadjuvant therapy.
