# Supplementary material for: Neoadjuvant Therapy for Extrahepatic Biliary Tract Cancer: A Propensity Score-Matched Survival Analysis
Source: J Clin Med. 2023 Apr 2;12(7):2654. doi: 10.3390/jcm12072654 (PMC10095506; doi:10.3390/jcm12072654)
Supplement: Supplementary file 1 [file jcm-12-02654-s001.zip › jcm-2247187-supplementary.pdf]

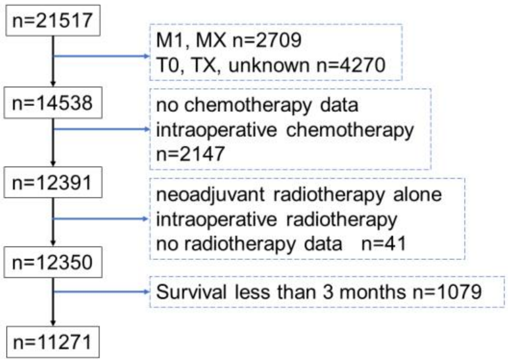

Supplemental Figure S1. Flowchart for selection of patients with extrahepatic bile tract cancer included in the study cohort.

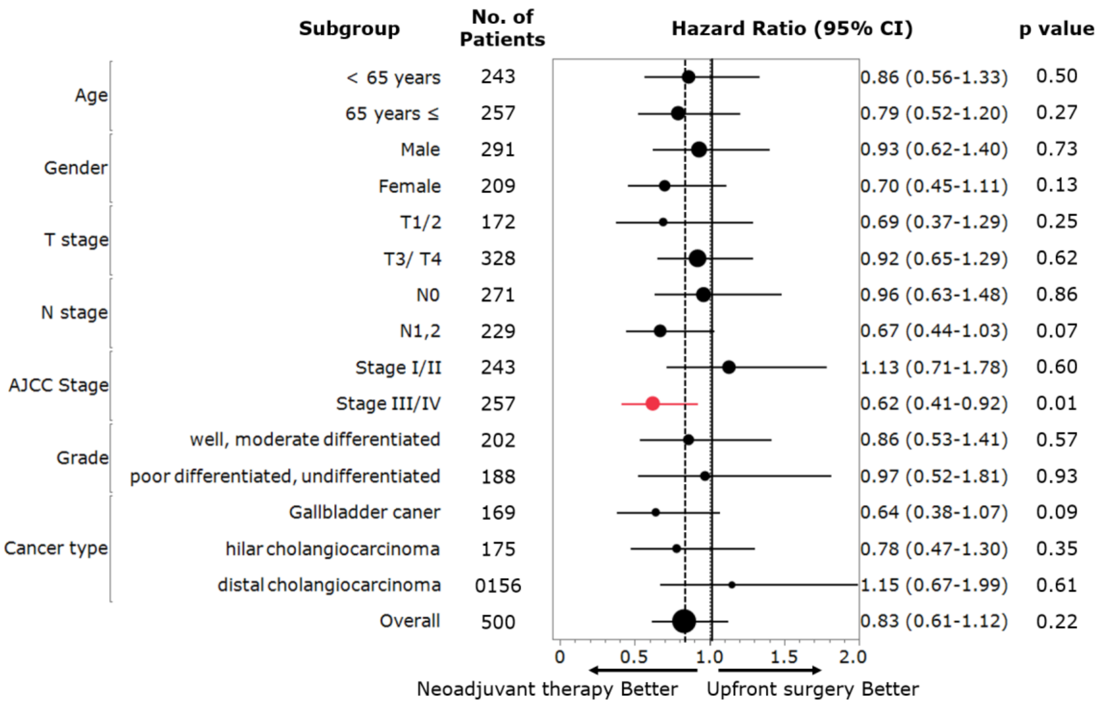

Supplemental Figure S2. Subgroup analyses of cancer-specific survival in the PSM

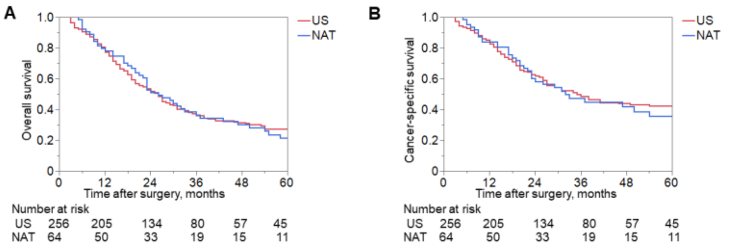

population.

Supplemental Figure S3. Kaplan–Meier curves depicting overall (A) and cancer-specific survival (B) stratified by use of NAT among patients with extrahepatic cholangiocarcinomas.

Supplemental Table S1. Univariate and multivariate Cox regression analyses with OS and CSS among patients with extrahepatic biliary tract cancer before propensity score matching.

	Univariate analysis	Multivariate analysis

Variables	Reference	HR	95%CI	p	HR	95%CI	p
**OS**							
Age ≥ 65	< 65	1.40	1.31-1.49	< 0.01	1.44	1.35-1.53	< 0.01
Male	Female	1.06	0.87-0.98	< 0.01	1.06	1.00-1.12	0.06
White	Other	1.08	1.01-1.16	0.02	1.04	0.97-1.11	0.25
GBC	EHCC	0.88	0.83-0.94	< 0. 01	1.12	1.04-1.20	< 0.01
Grade poor, undifferentiated	Well, moderate	1.55	1.46-1.65	< 0.01	1.36	1.28-1.45	< 0.01
T3/4	T1/2	2.02	1.91-2.15	< 0.01	1.90	1.78-2.03	< 0.01
N1/2	N0	1.65	1.56-1.75	< 0.01	1.43	1.34-1.53	< 0.01
Stages III/IVA	Stages I/II	1.93	1.82-2.04	< 0.01	-	-	-
Neoadjuvant therapy	No	1.05	0.83-1.31	0.67	0.86	0.68-1.09	0.22
Adjuvant therapy	No	1.13	1.07-1.20	< 0.01	0.87	0.81-0.93	< 0.01
**CSS**							
Age ≥ 65	< 65	1.13	1.05-1.22	< 0.01	1.20	1.19-1.29	< 0.01
Male	Female	1.05	0.97-1.12	0.16	1.05	0.98-1.13	0.15
White	Other	1.01	0.93-1.10	0.65	0.97	0.90-1.06	0.6
GBC	EHCC	0.92	0.86-1.00	0.04	1.26	1.16-1.36	< 0.01
Grade poor, undifferentiated	Well, moderate	1.74	1.61-1.87	< 0.01	1.47	1.37-1.58	< 0.01
T3/4	T1/2	2.49	2.32-2.67	< 0.01	2.28	2.11-2.46	< 0.01
N1/2	N0	1.86	1.73-2.00	< 0.01	1.47	1.36-1.59	< 0.01
Stages III/IVA	Stages I/II	2.45	2.28-2.62	< 0.01	-	-	-
Neoadjuvant therapy	No	1.06	0.82-1.39	0.62	0.84	0.64-1.10	0.21
Adjuvant therapy	No	1.35	1.26-1.45	< 0.01	0.95	0.88-1.03	0.28

OS: overall survival; CSS: cancer specific survival; GBC: gallbladder cancer; EHCC: extrahepatic cholangiocarcinoma.

Supplemental Table S2. Patient characteristics after propensity score matching stratified by neoadjuvant therapy.

	Upfront surgery		Neoadjuvant therapy		
Variable	n = 400	%, IQR	n = 100	%, IQR	p value
Age, n (%)					0.72
< 65 years	196	49.0	47	47.0	
≥ 65 years	204	51.0	53	53.0	
Gender, n (%)					0.34
Male	237	59.2	54	54.0	
Female	163	40.8	46	46.0	
Race, n (%)					0.41
White	326	81.5	85	85.0	
Others	74	18.5	15	15.0	
Type of cancer, n (%)					0.61
Gallbladder cancer	131	32.7	38	38.0	
Hilar cholangiocarcinoma	142	35.5	33	33.0	
Distal cholangiocarcinoma	127	31.8	29	18.0	
Year of diagnosis, n (%)					0.92
2006–2009	91	22.7	21	21.0	
2010–2013	145	36.3	38	38.0	
2014–2017	164	41.0	41	41.0	
AJCC T stage, n (%)					0.54
T1/2	135	33.8	37	37.0	
T3/4	265	66.2	63	63.0	
AJCC N stage, n (%)					0.35
N0	221	55.3	50	50.0	
N1/2	179	33.7	50	50.0	
AJCC stage, n (%)					
Stages I/II	199	49.8	44	44.0	
Stages III/IVA	201	51.2	56	56.0	
Tumor grade, n (%)					0.51
Well, moderate	162	40.5	40	40.0	
Poor, undifferentiated	154	38.5	34	34.0	
Unknown	84	21.0	26	26.0	
Adjuvant therapy, n (%)	207	51.8	46	46.0	

IQR: interquartile range; AJCC: American Joint Committee on Cancer.

Supplemental Table S3. Patient characteristics after propensity score matching among patients with stages III/IVA of the disease.

Variable	Upfront surgery		Neoadjuvant therapy		
	n = 201	%, IQR	n = 56	%, IQR	p value
Age, n (%)					0.94
< 65 years	98	48.8	27	48.2	
≥ 65 years	103	51.2	29	51.8	
Gender, n (%)					0.57
Male	109	54.2	28	50.0	
Female	92	45.8	28	50.0	
Race, n (%)					0.87
White	174	86.6	48	85.7	
Others	27	13.4	8	14.3	
Type of cancer, n (%)					0.18
Gallbladder cancer	99	49.2	33	58.9	
Hilar cholangiocarcinoma	97	48.3	20	35.7	
Distal cholangiocarcinoma	5	2.5	3	5.4	
Year of diagnosis, n (%)					0.21
2006–2009	71	35.3	15	26.8	
2010–2013	49	24.4	20	35.7	
2014–2017	81	40.3	21	37.5	
AJCC T stage, n (%)					0.87
T1/2	27	13.4	8	14.3	
T3/4	174	86.6	48	85.7	
AJCC N stage, n (%)					0.53
N0	81	40.3	20	35.7	
N1/2	120	59.7	36	64.3	
Tumor grade, n (%)					0.93
Well, moderate	77	38.3	23	41.1	
Poor, undifferentiated	79	39.3	21	37.5	
Unknown	45	22.4	12	21.4	
Adjuvant therapy, n (%)	108	53.7	23	41.1	0.09

IQR: interquartile range; AJCC: American Joint Committee on Cancer.

Supplemental Table S4. ECC patient characteristics after propensity score matching stratified by neoadjuvant therapy.

Variable	Upfront surgery		Neoadjuvant therapy		
	n = 256	%, IQR	n = 64	%, IQR	p value
Age, n (%)					0.73
< 65 years	130	50.8	31	48.4	
≥ 65 years	126	49.2	33	51.6	
Gender, n (%)					0.25
Male	183	71.5	41	64.1	
Female	73	28.5	23	35.9	
Race, n (%)					0.52
White	216	84.4	56	87.5	
Others	40	15.6	8	12.5	
Type of cancer, n (%)					0.46
Hilar cholangiocarcinoma	149	58.2	34	53.1	
Distal cholangiocarcinoma	107	41.8	30	46.9	
Year of diagnosis, n (%)					0.94
2006–2009	51	19.9	13	20.3	
2010–2013	95	37.1	25	39.1	
2014–2017	110	43.0	26	40.6	
AJCC T stage, n (%)					0.69
T1/2	109	42.6	29	45.3	
T3/4	147	57.4	35	54.7	
AJCC N stage, n (%)					0.37
N0	128	50.0	36	56.2	
N1/2	128	50.0	28	43.8	
AJCC Stage, n (%)					0.86
Stages I/II	157	61.3	40	62.5	
Stages III/IVA	99	38.7	24	37.5	
Tumor grade, n (%)					0.45
Well, moderate	99	38.6	27	42.2	
Poor, undifferentiated	101	39.4	20	31.2	
Unknown	56	21.8	17	26.6	
Adjuvant therapy, n (%)	134	52.3	35	54.7	0.73
